# Supplementary material for: Iterative improvement in the automatic modular design of robot swarms
Source: PeerJ Comput Sci. 2020 Dec 7;6:e322. doi: 10.7717/peerj-cs.322 (PMC7924708; doi:10.7717/peerj-cs.322)
Supplement: Supplemental Information 3 [file peerj-cs-06-322-s003.zip › argos3/doc/api/standalone/a00446_source.html]

ARGoS: plugins/robots/foot-bot/control\_interface/ci\_footbot\_motor\_ground\_sensor.cpp Source File


- Main Page
- Related Pages
- Namespaces
- Classes
- Files

- File List
- File Members

# plugins/robots/foot-bot/control\_interface/ci\_footbot\_motor\_ground\_sensor.cpp

Go to the documentation of this file.

```
00001 
00008 #include "ci_footbot_motor_ground_sensor.h"
00009 
00010 #ifdef ARGOS_WITH_LUA
00011 #include <argos3/core/wrappers/lua/lua_utility.h>
00012 #endif
00013 
00014 namespace argos {
00015 
00016    /****************************************/
00017    /****************************************/
00018 
00019    CCI_FootBotMotorGroundSensor::CCI_FootBotMotorGroundSensor() :
00020       m_tReadings(4) {
00021       // Set the values for the motor ground sensor offset (taken from the CAD model, in cm)
00022       m_tReadings[0].Offset.Set( 6.3, 1.16);
00023       m_tReadings[1].Offset.Set(-6.3, 1.16);
00024       m_tReadings[2].Offset.Set(-6.3,-1.16);
00025       m_tReadings[3].Offset.Set( 6.3,-1.16);
00026    }
00027     
00028    /****************************************/
00029    /****************************************/
00030 
00031    const CCI_FootBotMotorGroundSensor::TReadings& CCI_FootBotMotorGroundSensor::GetReadings() const {
00032      return m_tReadings;
00033    }
00034 
00035    /****************************************/
00036    /****************************************/
00037 
00038    std::ostream& operator<<(std::ostream& c_os,
00039                             const CCI_FootBotMotorGroundSensor::SReading& s_reading) {
00040       c_os << "Value=<" << s_reading.Value
00041            << ">, Offset=<" << s_reading.Offset << ">";
00042       return c_os;
00043    }
00044 
00045    /****************************************/
00046    /****************************************/
00047    
00048    std::ostream& operator<<(std::ostream& c_os,
00049                             const CCI_FootBotMotorGroundSensor::TReadings& t_readings) {
00050       if(! t_readings.empty()) {
00051          c_os << "{ " << t_readings[0].Value << " }";
00052          for(UInt32 i = 1; i < t_readings.size(); ++i) {
00053             c_os << " { " << t_readings[0].Value << " }";
00054          }
00055          c_os << std::endl;
00056       }
00057       return c_os;
00058    }
00059 
00060    /****************************************/
00061    /****************************************/
00062    
00063 #ifdef ARGOS_WITH_LUA
00064    void CCI_FootBotMotorGroundSensor::CreateLuaState(lua_State* pt_lua_state) {
00065       CLuaUtility::OpenRobotStateTable(pt_lua_state, "motor_ground");
00066       for(size_t i = 0; i < m_tReadings.size(); ++i) {
00067          CLuaUtility::StartTable(pt_lua_state, i+1                            );
00068          CLuaUtility::AddToTable(pt_lua_state, "offset", m_tReadings[i].Offset);
00069          CLuaUtility::AddToTable(pt_lua_state, "value",  m_tReadings[i].Value );
00070          CLuaUtility::EndTable  (pt_lua_state                                 );
00071       }
00072       CLuaUtility::CloseRobotStateTable(pt_lua_state);
00073    }
00074 #endif
00075 
00076    /****************************************/
00077    /****************************************/
00078 
00079 #ifdef ARGOS_WITH_LUA
00080    void CCI_FootBotMotorGroundSensor::ReadingsToLuaState(lua_State* pt_lua_state) {
00081       lua_getfield(pt_lua_state, -1, "motor_ground");
00082       for(size_t i = 0; i < m_tReadings.size(); ++i) {
00083          lua_pushnumber(pt_lua_state, i+1                 );
00084          lua_gettable  (pt_lua_state, -2                  );
00085          lua_pushnumber(pt_lua_state, m_tReadings[i].Value);
00086          lua_setfield  (pt_lua_state, -2, "value"         );
00087          lua_pop       (pt_lua_state, 1                   );
00088       }
00089       lua_pop(pt_lua_state, 1);
00090    }
00091 #endif
00092 
00093 
00094    /****************************************/
00095    /****************************************/
00096 
00097 }
```

---

Generated on 10 Jul 2018 for ARGoS by 
 1.6.1
